# All-cause 30- and 90-day inpatient readmission costs associated with 4 minimally invasive colon surgery approaches: A propensity-matched analysis using Medicare and commercial claims data

**DOI:** 10.1016/j.sopen.2022.09.007

**Published:** 2022-09-25

**Authors:** Michelle P. Sosa, Deirdre G. McNicholas, Arbelina B. Bebla, Keith A. Needham, Paul M. Starker

**Affiliations:** a Stryker Endoscopy, San Jose, CA; b Baker Tilly US, LLP, New York, NY, USA; c Overlook Medical Center, Columbia University Vagelos College of Physicians and Surgeons, New York, NY, USA

**Keywords:** CMS, Centers for Medicare & Medicaid Services, HAC, Hospital-Acquired Condition Reduction Program, ICD-10-CM, International Classification of Diseases, 10th Revision, Clinical Modification, ICD-10-PCS, International Classification of Diseases, 10th Version, Procedure Coding System, IP, inpatient, MIS, minimally invasive surgery, NIF, near-infrared fluorescence, R, robotics

## Abstract

**Background:**

The purpose of this study is to assess which minimally invasive colon surgery approach may be associated with the least 30- and 90-day inpatient readmission costs from a payer perspective.

**Methods:**

This retrospective claims analysis included adult Medicare and commercially insured beneficiaries who underwent minimally invasive sigmoid, left, or right colon surgery between January 2016 and December 2019. Two cohorts were created based on the use of near-infrared fluorescence (NIF) and were propensity-score matched 1 NIF:5 NoNIF. Four subgroups were then created based on the presence of robotics (R): NIF-NoR, NIF-R, NoNIF-R, and NoNIF-NoR.

**Results:**

A total of 50,148 patients were identified, of which 165 (0.3%) indicated the use of NIF and 49,983 (99.7%) did not. After propensity score matching, 990 patients were included (NIF cohort: 165; NoNIF cohort: 825). Of the 165 NIF patients, 87 were robotic-assisted and 78 were conventional laparoscopy. Of the 825 NoNIF patients, 136 were robotic-assisted and 689 were conventional laparoscopy. Postindex inpatient readmission costs were significantly different between the NIF and NoNIF cohorts with the NIF cohort having the lowest 30- and 90-day postindex readmission costs. Postindex readmission costs were also significantly different across the 4 subgroups at 30 and 90 days, with the NIF-NoR group having the lowest postindex readmission costs (all P < .05).

**Conclusion:**

Using NIF without the robot during minimally invasive colon surgery is associated with the least 30- and 90-day inpatient readmission costs compared to the other 3 approaches. Hospitals may want to consider these potential cost savings when evaluating technologies for laparoscopic colon surgery.

**Key Message:**

Near-infrared fluorescence (NIF) imaging without the robot during minimally invasive colon surgery may significantly save hospitals 30- and 90-day inpatient readmission costs compared to NIF with the robot, NoNIF with the robot, and NoNIF without the robot. This is important as hospitals may want to consider these cost findings in addition to capital equipment and disposable costs when evaluating technologies for laparoscopic colon surgery.

## INTRODUCTION

Hospital readmissions impose a significant burden on patients and health care systems and are an important metric for assessing performance and quality of patient care. The Healthcare Cost and Utilization Project reports that, in 2018, nearly 14% of adults in the United States were readmitted within 30 days after hospital discharge with an average readmission cost of $15,200 [1]. Readmission within 30 days is common among colorectal surgery patients, ranging from 5.1% to 13.7% (*P* < .01), and is associated with a cost of approximately $9,000 per readmission [[2], [3], [4], [5], [6], [7]]. Studies report 90-day readmissions rates of up to approximately 24% [2,8]. Therefore, reducing 30- and 90-day hospital readmission rates and expenditures is an opportunity for both quality improvement and cost control.

A systematic review including 34 studies reports that postoperative complications are associated with a greater incidence of hospital readmission and that surgical site infections and anastomotic leaks are associated with greater resource utilization relative to other postoperative complications following colon resection surgery [9]. Anastomotic leaks have serious clinical-economic and health care utilization implications. This complication has a reported incidence between 1.5% and 23% and leads to significant morbidity and mortality, longer hospital stays, and significantly increased cost of care [[10], [11], [12], [13]]. These findings reinforce a previous claims analysis of 99,879 records which determined that patients with anastomotic leaks had 1.3 times higher 30-day readmission rates and incurred additional average length of stay increases of 7.3 days and additional hospital costs of $24,129 for hospitalization alone compared to patients without anastomotic leaks (*P* < .001 for all) [14]. It is important to note that anastomotic leaks typically appear within 30 days of a patient's initial surgery; however, nearly half of all leaks occur after the patient has been discharged, with up to 12% occurring after 30 days postindex hospitalization [[15], [16], [17], [18]]. Adequate blood supply and perfusion of the anastomosis are key for optimal anastomotic healing [19,20]. Therefore, detection of poor blood supply and perfusion during surgery may potentially reduce the occurrence of this complication [21,22].

Evidence suggests that intraoperative near-infrared fluorescence imaging using indocyanine green may reduce the incidence of anastomotic complications such as strictures and leaks without prolonging operation time or increasing postoperative complications [[22], [23], [24], [25], [26], [27], [28], [29], [30], [31], [32], [33], [34], [35]]. Numerous publications report that this technique enables improved visualization, allows confirmation of adequate perfusion of the anastomosis, and empowers surgeons to more accurately determine whether a change in the resection margin is warranted in an effort to significantly reduce costly postindex anastomotic failures [20,[28], [29], [30], [31], [32], [33], [34], [35], [36], [37], [38], [39], [40]]. For example, a recent systematic review and meta-analysis including 32 studies involving 11,047 patients revealed a lower incidence of anastomotic leaks in cases with fluorescence use (3.7% vs 7.6%, *P* < .001) [10]. Additionally, a recently published cost analysis determined the routine use of intraoperative near-infrared fluorescence imaging using indocyanine green to be cost saving [41]. Because of these reasons, the utilization of near-infrared fluorescence imaging using indocyanine green during minimally invasive surgery continues to rise.

Colon surgery is a common procedure with a high rate of postoperative adverse events that are expected to substantially contribute to hospital costs [9]. Studies comparing conventional laparoscopic and robotic-assisted minimally invasive colon surgery show that both techniques are safe, feasible, and demonstrate comparable outcomes, but that robotic-assisted surgery significantly increases costs [[42], [43], [44], [45], [46]]. To our knowledge, there are currently no studies that assess which minimally invasive colon surgery approach may be associated with the least 30- and 90-day downstream complication and inpatient hospital readmission rates and costs. Understanding which approach may optimize improved patient outcomes and consequently help to reduce costs would be beneficial for health care providers with limited financial resources.

## MATERIALS AND METHODS

### Study Design and Data Sources

This is a retrospective claims analysis of patients insured by Medicare or a single nationwide commercial payer undergoing inpatient laparoscopic-assisted endoscopic procedures commonly referred to as *minimally invasive surgery* from the payer perspective. We obtained claims-level Medicare data for these patients from the Center for Medicare & Medicaid Services (CMS)-compiled Medicare 100% Standard Analytical Files, which are representative of medical services provided to nearly 37 million Medicare Fee-for-Service beneficiaries. To evaluate commercial payer trends, we used data from the OptumInsight Inc (Eden Prairie, MN) database, which is representative of claims from roughly 25 million members of a nationwide United States–based health insurance plan. We chose a 3-year follow-up period to examine outcomes and utilization extending beyond the immediate postoperative period.

### Inclusion Criteria

Adult Medicare and commercial beneficiaries who received a minimally invasive sigmoid, left, or right colon surgery in an inpatient setting between January 1, 2016, and December 31, 2019 (referred to as the *index period*), were included and identified by the presence of ICD-10-PCS codes describing excision and resection procedures (see Appendix A). This time period was specifically selected to optimize the sample size because CMS established the near-infrared fluorescence code, ICD-10-PCS 4A1BXSH (Monitoring of Gastrointestinal Vascular Perfusion using Indocyanine Green Dye, External Approach), in 2016.

### Exclusion Criteria

Patients with malignant neoplasm of rectum (ICD-10-CM Diagnosis Code C20) or secondary malignant neoplasm of large intestine and rectum (ICD-10-CM Diagnosis Code C78.5) present at index or who had missing cost information for index procedure were excluded.

### Four Minimally Invasive Surgery Patient Groups

Following the identification of MIS claims, 2 study cohorts were created: MIS using near-infrared fluorescence (NIF) imaging and MIS without NIF (NoNIF) imaging. NIF cases required an ICD-10-PCS code indicative of an NIF procedure (see Appendix A). The sample was further divided to create a total of 4 subgroups based on the use of NIF and robotics (R), as follows:

1. Robotic-assisted MIS with NIF (NIF-R)
2. Robotic-assisted MIS without NIF (NoNIF-R)
3. Conventional MIS with NIF (NIF-NoR)
4. Conventional MIS without NIF (NoNIF-NoR)

**Fig 1:**
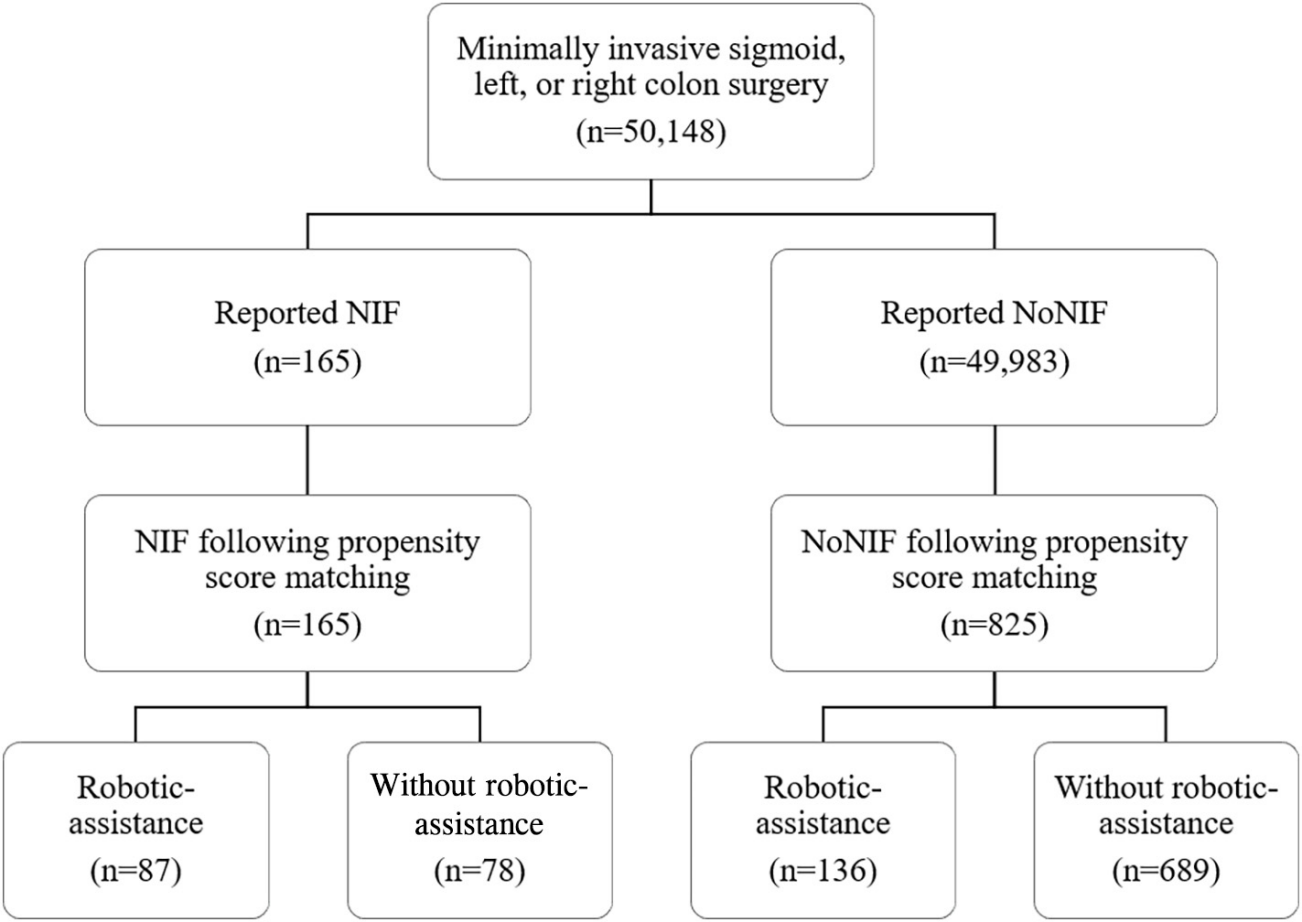
This flowchart shows how many patients were included in the study.

Robotic-assisted cases required an ICD-10-PCS code indicative of a robotic-assisted procedure (see Appendix A). Cases in the conventional cohorts did not have the ICD-10-PCS robotic-assisted procedure code. A detailed flowchart of the case selection methodology is illustrated in Fig 1.

### Outcomes

We assessed downstream complication and all-cause inpatient readmission rates and costs at 30-day and 90-day postindex intervals.

### Costs

Cost estimates used in this analysis represent total payments to providers made by CMS and a single national commercial payer for those who underwent minimally invasive colon surgery.

### Downstream Complications

Complication categories included revision, dehiscence, infections, stricture, accidental puncture, shock, urinary tract infection, stoma, and other. A claim with codes identifying adverse events from any of these categories is broadly classified as experiencing a downstream complication. See Appendix B for the ICD-10-CM and ICD-10-PCS codes used to identify downstream complications.

### Propensity Score Matching

We performed a propensity score–matched analysis in anticipation of smaller counts for groups reporting the use of indocyanine green and conducted Pearson *χ^2^* tests to examine baseline differences in demographic and clinical characteristics. A 1:5 propensity score match was conducted to ensure a balanced sample between the cohorts. Each patient in the NIF cohort was matched to 5 patients in the NoNIF cohort. NIF patients were matched to NoNIF patients to address the differences in age, sex, and high-cost comorbidities (chronic obstructive pulmonary disease, hypertension, diabetes, coronary artery disease, smoking, cancer, and obesity).

### Statistical Analysis

We used a stepwise approach to analyze subgroup differences. First, we analyzed the NIF cohort versus the NoNIF cohort. Then, we analyzed the 4 subgroups for group differences. Pearson *χ^2^* tests were used to test cohort differences in the probability of having costs in the 30- and 90-day postindex periods. Mann–Whitney *U* and analysis of variance tests were used to analyze cohort differences in cost during the same 30- and 90-day postindex time periods. The Mann–Whitney *U* test was used when comparing 2 groups (NIF vs NoNIF), and the analysis of variance test was used when comparing differences among more than 2 groups (i.e., the 4 MIS subgroups). All propensity score matching and analyses were performed using SAS Enterprise Guide 7.1 software (SAS Institute Inc, Cary, NC).

## RESULTS

### Patient Population

A total of 50,148 subjects had a claim for receiving a minimally invasive sigmoid, left, or right colon surgery during the study period (January 1, 2016 through December 31, 2019). From this population, 165 (.3%) claims indicated the use of NIF and 49,983 (99.7%) did not indicate use of NIF. The 1 to 5 propensity score–matched analysis resulted in 990 patients (165 patients treated with NIF and 825 not treated with NIF). Of the 165 NIF patients, 87 were robotic-assisted and 78 were conventional laparoscopic. Of the 825 NoNIF patients, 136 were robotic-assisted and 689 were conventional laparoscopic (Fig 1). Prior to matching the NIF and NoNIF subjects on age, sex, and high-cost comorbidities, the NIF population had significantly lower cases of diabetes compared to the NoNIF group (*P* = .006) (Table 1). The postmatch index analyses stratified for NIF show comparable characteristics across all variables (Table 1). The postmatch index analyses stratified for the 4 subgroups also show comparable characteristics across all variables (Table 2).

**Table 1:** Pre- versus postmatched index patient characteristics

	*Before propensity score matching*			*After propensity score matching*		
	*NIF* *(*n *= 165)*	*NoNIF* *(*n *= 49,983)*	P	*NIF* *(*n *= 165)*	*NoNIF* *(*n *= 825)*	P
	n *(%)*	n *(%)*		n *(%)*	n *(%)*	
Age			.329			.999
< 65	43 (26.1)	12,231 (24.5)		43 (26.1)	216 (26.2)	
65–69	35 (21.2)	11,381 (22.8)		35 (21.2)	178 (21.6)	
70–74	33 (20.0)	9502 (19.0)		33 (20.0)	165 (20.0)	
75–79	28 (17.0)	7582 (15.2)		28 (17.0)	139 (16.9)	
80–84	20 (12.1)	5098 (10.2)		20 (12.1)	97 (11.8)	
> 84	< 11†	4184 (8.4)		< 11†	30 (3.6)	
Sex						
Male	79 (47.9)	21,938 (43.9)	.303	79 (47.9)	399 (48.4)	.909
High-cost comorbidities						
COPD	14 (8.5)	5268 (10.5)	.392	14 (8.5)	67 (8.1)	.876
Smoking	23 (13.9)	4768 (9.5)	.055	23 (13.9)	112 (13.6)	.901
Hypertension	79 (47.9)	24,797 (49.6)	.657	79 (47.9)	397 (48.1)	.955
Diabetes	23 (13.9)	11,461 (22.9)	.006⁎	23 (13.9)	112 (13.6)	.901
CAD	21(12.7)	8032 (16.1)	.243	21 (12.7)	111 (13.5)	.802
Oncology	72 (43.6)	19,908 (39.8)	.319	72 (43.6)	358 (43.4)	.954
Obesity	19 (11.5)	7812 (15.6)	.147	19 (11.5)	93 (11.3)	.929

Categorical variables reported as count (percentage).

*CAD, coronary artery disease; COPD, chronic obstructive pulmonary disease*.

⁎ Designates significance (*P* < .05).

† Low patient counts have been removed and replaced with "< 11" because of a data use agreement.

**Table 2:** Postmatch index patient characteristics stratified by NIF and robotic assistance.

	*NIF-R* *(*n *= 87)*	*NoNIF-R* *(*n *= 136)*	*NIF-NoR* *(*n *= 78)*	*NoNIF-NoR* *(*n *= 689)*	P
	n *(%)*	n *(%)*	n *(%)*	n *(%)*	
Age					.881
< 65	17 (19.5)	33 (24.3)	26 (33.3)	183 (26.6)	
65–69	18 (20.7)	28 (20.6)	17 (21.8)	150 (21.7)	
70–74	21 (24.1)	35 (25.7)	12 (15.4)	130 (18.9)	
75–79	17 (19.5)	22 (16.2)	11 (14.1)	117 (17.0)	
80–84	11 (12.6)	14 (10.3)	< 11†	83 (12.1)	
> 84	< 11†	< 11†	< 11†	26 (3.8)	
Sex					
Male	34 (39.1)	66 (48.5)	45 (57.7)	333 (48.3)	.126
High-cost comorbidities					
COPD	< 11†	11 (8.1)	< 11†	56 (8.1)	.986
Smoking	11 (12.6)	17 (12.5)	12 (15.4)	95 (13.8)	.932
Hypertension	48 (55.2)	75 (55.2)	31 (39.7)	322 (46.7)	.067
Diabetes	< 11⁎	23 (16.9)	13 (16.7)	89 (12.9)	.477
CAD	< 11⁎	20 (14.7)	13 (16.7)	91 (13.2)	.518
Oncology	36 (41.4)	64 (47.1)	36 (46.2)	294 (42.7)	.735
Obesity	< 11†	13 (9.6)	< 11†	80 (11.6)	.922
Surgical approach					
Left	< 11†	< 11†	< 11†	28 (4)	.183
Right	54 (62)	65 (48)	44 (56)	199 (29)	.0004⁎

Categorical variables reported as count (percentage).

⁎ Designates significance (*P* < .05).

† Low patient counts have been removed and replaced with "< 11" because of a data use agreement.

### Downstream Complications

The downstream complication rates and costs for the NIF and NoNIF cohorts are shown in Table 3. The downstream complication rates and costs for the NIF cohort and NoNIF cohort at 30 and 90 days were comparable. Subgroup results are not included for this outcome because of lower patient counts (< 11) experiencing complications across the 4 subgroups.

**Table 3:** Downstream complication rates and costs by NIF cohort

	*NIF* *(*n *= 165)*		*NoNIF* *(*n *= 825)*		*Decrease*⁎*(%)*	P
Rates	% (*n*)	95% CI	% (*n*)	95% CI		
30 d	6.7 (11)	.092–.295	6.2 (51)	.094–.168	7.5	.261
90 d	9.1 (15)	.118–.308	9.5 (78)	.142–.219	− 4.4	.927
Costs	$ ± SD	95% CI	$ ± SD	95% CI		
30 d	$21,856 ± $32,997	$45–$41,976	$77,775 ± $258,504	$0–$157,424	− 255.9	.446
90 d	$37,259 ± $57,640	$4948–$66,376	$63,152 ± $202,843	$13,178–$109,911	− 69.5	.614

Categorical variables reported as percentage (count). Costs reported in United States dollars.

*CI*, confidence interval; *SD*, standard deviation.

⁎ Values represent percentage decrease between NIF and NoNIF cases.

### Postindex Services: Inpatient Readmissions

The inpatient (IP) readmission rates and costs for the NIF and NoNIF cohorts are shown in Table 4. The differences in IP readmission rates at 30 and 90 days were not statistically significant between the NIF and NoNIF cohorts. However, IP readmission costs at both time periods were significantly lower for the NIF cohort than the NoNIF cohort. At 30 days, IP readmission costs were 74.5% lower for the NIF cohort than the NoNIF cohort (*P* = .020). At 90 days, IP readmission costs were 45.1% lower for the NIF cohort than the NoNIF cohort (*P* = .048).

**Table 4:** Inpatient readmission rates and average costs by NIF cohort

	*NIF* *(*n *= 165)*		*NoNIF* *(*n *= 825)*		*Decrease*† *(%)*	*p*
Rates	% (*n*)	95% CI	% (*n*)	95% CI		
30 d	13.9 (23)	.953–1.134	13.1 (108)	.991–1.028	5.8	.769
90 d	21.8 (36)	.977–1.134	22.8 (188)	.996–1.068	− 4.6	.786
Costs	$ ± SD	95% CI	$ ± SD	95% CI		
30 d	$26,354 ± $41,581	$8373–$44,335	$45,990 ± $164,438	$14,623–$77,358	− 74.5	.020⁎
90 d	$37,255 ± $69,426	$13,764–$60,745	$54,066 ± $179,852	$28,190–$79,943	− 45.1	.048⁎

Categorical variables reported as percentage (count). Costs reported in United States dollars.

⁎ Designates significance (*P* < .05).

† Values represent percentage decrease between NIF and NoNIF cases.

The IP readmission rates and costs for the 4 subgroups are shown in Table 5. When performing the subgroup analyses, we found significantly different utilization rates at 90 days across all 4 subgroups, with the NoNIF-NoR subgroup having the highest IP readmission rate at 24.8% (*P* = .018). The 4 subgroups also differed significantly in IP readmission costs at 30 and 90 days (*P* = .024 and .040, respectively), with the NIF-NoR subgroup, conversely, having the lowest costs across all 4 groups at 5 times lower costs than the NIF-R subgroup, 2 to 3 times lower costs than the NoNIF-R subgroup, and 4 to 6 times lower costs than the NoNIF-NoR subgroup.

**Table 5:** Postoperative inpatient readmission rates and average costs stratified by NIF and robotic assistance

	*NIF-R* *(*n *= 87)*	*NoNIF-R* *(*n *= 136)*	*NIF-NoR* *(*n *= 78)*	*NoNIF-NoR* *(*n *= 689)*	P
Rates	% (*n*)	% (*n*)	% (*n*)	% (*n*)	
30 d	13.8 (12)	8.1 (11)	14.1 (11)	14.1 (97)	.303
90 d	20.7 (18)	12.5 (17)	23.1 (18)	24.8 (171)	.018⁎
Costs	$ ± SD	$ ± SD	$ ± SD	$ ± SD	
30 d	$42,444 ± $53,320	$22,522 ± $18,629	$8802 ± $5199	$48,652 ± $173,297	.024⁎
90 d	$61,568 ± $92,666	$27,461 ± $23,097	$12,941 ± $9200	$56,711 ± $188,291	.040⁎

Categorical variables reported as percentage (count). Costs reported in United States dollars.

## DISCUSSION

Several studies have shown that robotic-assisted colon surgery is associated with a significantly higher cost of care compared to conventional laparoscopy [[42], [43], [44], [45], [46]], predominantly due to its longer procedure times [44,45]. A propensity score–matched analysis using a nationwide hospital record database including 531,536 patients undergoing surgical treatment for colon cancer determined that robotic-assisted surgery was associated with a 13.6% higher cost than laparoscopic surgery (*P* < .0001) [42]. Similarly, a randomized clinical trial of robotic-assisted versus standard laparoscopic right colectomy found that duration of surgery was 50% longer in the robotic-assisted group (*P* < .001), and total hospital costs were 18.6% higher for the robotic-assisted group (*P* = .013), which were attributed primarily to the 48.6% higher cost of surgery and consumables for the robotic-assisted group (*P* < .001) [44]. These figures do not factor in the $1.55 million investment for the robot and its annual service contract ranging from $80,000 to $190,000 [47,48]. Adoption of robotic-assisted surgery is trending higher than conventional laparoscopic surgery despite its higher cost and comparable outcomes to conventional laparoscopic surgery [[42], [43], [44], [45], [46],^49^].

In this study, the downstream complication costs were 3.6 times lower at 30 days and 1.7 times lower at 90 days for the NIF cohort compared to the NoNIF cohort, albeit not significantly lower (Table 3). Reduced complication costs for the NIF cohort were also seen in a study of 347 patients undergoing colectomy with primary anastomosis [39]. In that study, those assessed with NIF experienced an 84.7% reduction in anastomotic failures, which corresponded to a 7.6% reduction in average cost per case per 100 cases [39]. Although the downstream complications data for the 4 subgroups were not shown here due to lower patient counts, the following average revision costs were observed: $0 for NIF-NoR, $11,664 for NoNIF-R, $29,653 for NIF-R, and $63,637 for NoNIF-NoR. Of the 4 approaches, the NIF-NoR subgroup reported no revisions at 30 days postindex hospitalization and therefore demonstrated the lowest revision costs. The other 3 subgroups experienced at least 1 or more revisions during the same time period.

Additionally, the postindex readmission costs were 1.7 times lower at 30 days and 1.5 times lower at 90 days for the NIF cohort compared to the NoNIF cohort (Table 4). A plausible explanation is that those in the NoNIF cohort were readmitted for more severe complications which may have been indicative of an anastomotic failure because severe complications cost more to treat. Similarly, the 30- and 90-day post-index readmission costs were significantly different across the 4 subgroups, with NIF-NoR demonstrating the least readmission costs at both time intervals (Table 5). This suggests that patients treated with NIF-NoR experienced the least number of complications or experienced less severe complications among the 4 approaches. These findings support studies which demonstrate a reduction in anastomotic complications in patients treated with NIF-NoR resulting from a change in surgical plan based on intraoperative perfusion assessment with fluorescence angiography [[30], [31], [32], [33], [34], [35], [36], [37]]. Some of these studies observed anastomotic complication reductions ranging from 59% to 90% among those intraoperatively assessed with NIF-NoR [35,36].

Although this study was performed from the payer perspective using total payments made to providers to estimate cost, payments serve as a reasonable estimation of facility costs. For example, Medicare acknowledges that procedures resulting in more severe complications incur higher costs than procedures resulting in less severe or no complications by providing higher reimbursement for the former [50]. Because postoperative complications may contribute to higher costs, lower postindex inpatient readmission costs may be indicative of providing better quality care for patients.

Providing better quality of care is advocated by CMS' ongoing value-based initiatives such as the Hospital Readmissions Reduction Program and the Hospital-Acquired Condition (HAC) Reduction Program. Both programs help to facilitate the transition of the United States health care system from a fee-for-service system to a value-based system [51]. The Hospital Readmissions Reduction Program is a value-based purchasing program established by CMS to combat excessive and costly readmissions by reducing a hospital's overall reimbursement up to 3% for excessive readmission rates for certain diagnoses including acute myocardial infarction, chronic obstructive pulmonary disease, heart failure, pneumonia, coronary artery bypass graft surgery, elective primary total hip arthroplasty, and/or total knee arthroplasty [52]. Therefore, hospital readmission rates are recognized as a major quality and cost-containment metric for hospitals, clinicians, and policy makers. Additionally, the HAC Reduction Program seeks to ameliorate patient safety and lower the occurrence of hospital-acquired conditions, including but not limited to postoperative wound dehiscence, colon surgical site infections, and unrecognized abdominopelvic accidental puncture/lacerations, by penalizing hospitals categorized in the worst performing quartile with a 1% reduction in payments [53]. As the treatment of these HACs may contribute to inpatient readmission costs, our results suggest that health care providers using NIF-NoR may benefit from significantly lower postindex inpatient readmission costs at 30 and 90 days compared to the other 3 MIS approaches.

There are several limitations to this study. First, only 1 national commercial payer's database was available for this analysis. Second, our analysis did not account for the capital and disposable investment as well as maintenance costs which may potentially widen the cost differences. This was done because the objective was to analyze the economic impact to payers. Third, low procedural volumes for the robotic and NIF technology data sets were included in this study, which created challenges when identifying statistical significances between cohorts. The authors believe that the low volumes may be attributed to the underreporting of both the robotic and NIF ICD-10-PCS codes. Therefore, it is possible that some NoNIF cases may have been assessed with NIF.

Additionally, there are challenges to deriving insights from claims data using the appropriate codes to identify the patient populations of interest, their precise diagnosis at each time point, their clinical progression, and outcomes. We performed a propensity score–matched analysis to minimize confounding and account for varying baseline patient characteristics. Another important consideration is that many factors could have also affected whether a surgeon reported the use of a particular MIS technique at the time of their patients' care including the availability of NIF or the robot. However, NIF has been around for decades and widely used in numerous surgical specialties including colon surgery [54].

In conclusion, as hospitals seek to work within their financial constraints while striving for optimal patient outcomes, it is important to consider which MIS approach may most effectively improve patient outcomes and potentially help to reduce health care resource utilization and costs. This study demonstrates that implementing NIF imaging without the robot during MIS may significantly save hospitals 30- and 90-day inpatient readmissions costs compared to the other 3 MIS approaches. Hospitals may want to consider these potential cost savings in addition to capital equipment and disposable costs when evaluating technologies for minimally invasive colon surgery.

## Author Contribution

We verify that all listed authors substantially contributed to the manuscript warranting authorship.

## Funding Source

The 10.13039/100008894Stryker Corporation provided funding for this study.

## Ethics Approval

Not applicable.

^⁎^ Designates significance (*P* < .05).

## Conflict of Interest

Stryker Corporation employs Michelle Sosa, Deirdre McNicholas, and Arbelina Bebla. Dr Starker is a consultant to Stryker Corporation. Keith Needham has no conflict of interest to declare.

## Figures and Tables

**Table:** 

*Procedure*	*ICD-10-PCS code*
Endoscopy using laparoscopy (includes excision and resection procedures)	0D194Z9, 0D194ZA, 0D194ZB, 0D194ZL, 0D198Z9, 0D198ZA, 0D198ZB, 0D198ZL, 0D1A4ZA, 0D1A4ZB, 0D1A4ZH, 0D1A4ZK, 0D1A4ZL, 0D1A4ZM, 0D1A4ZN, 0D1A4ZP, 0D1A8ZA, 0D1A8ZB, 0D1A8ZH, 0D1A8ZK, 0D1A8ZL, 0D1A8ZM, 0D1A8ZN, 0D1A8ZP, 0D1B4ZB, 0D1B4ZH, 0D1B4ZL, 0D1B4ZM, 0D1B4ZN, 0D1B4ZP, 0D1B4ZQ, 0D1B8ZB, 0D1B8ZH, 0D1B8ZK, 0D1B8ZL, 0D1B8ZM, 0D1B8ZN, 0D1B8ZP, 0D1B8ZQ, 0D1H4ZH, 0D1H4ZK, 0D1H4ZL, 0D1H4ZM, 0D1H4ZN, 0D1H4ZP, 0D1H8ZH, 0D1H8ZK, 0D1H8ZL, 0D1H8ZM, D1H8ZN, 0D1H8ZP, 0D1K4ZK, 0D1K4ZL, 0D1K4ZM, 0D1K4ZN, 0D1K4ZP, 0D1K8ZK, 0D1K8ZL, 0D1K8ZM, 0D1K8ZN, 0D1K8ZP, 0D1L4Z4, 0D1L4ZL, 0D1L4ZM, 0D1L4ZN, 0D1L4ZP, 0D1L8ZL, 0D1L8ZM, 0D1L8ZN, 0D1L8ZP, 0D1M4Z4, 0D1M4ZM, 0D1M4ZN, 0D1M4ZP, 0D1M8ZM, 0D1M8ZN, 0D1M8ZP, 0D1N4Z4, 0D1N4ZN, 0D1N4ZP, 0D1N8ZN, 0D1N8ZP, 0DBE3ZZ, 0DBE4ZZ, 0DBE8ZZ, 0DBGFZZ, 0DBLFZZ, 0DBMFZZ, 0DBNFZZ, 0DTE4ZZ, 0DTE8ZZ, 0DTF4ZZ, 0DTF8ZZ, 0DTG4ZZ, 0DTG8ZZ, 0DTGFZZ, 0DTH4ZZ, 0DTH8ZZ, 0DTL4ZZ, 0DTL8ZZ, 0DTLFZZ, 0DTMFZZ, 0DTN4ZZ, 0DTN8ZZ, 0DTNFZZ
Near-infrared fluorescence	4A1BXSH (monitoring of gastrointestinal vascular perfusion using indocyanine green dye, external approach)
Robotic assistance	8E0W0CZ, 8E0W4CZ, 8E0W3CZ, 8E0W8CZ, 8E0W7CZ, 8E0WXCZ

*ICD-10-PCS*, International Classification of Diseases, 10th Revision, Procedure Coding System.

**Table:** 

*Category*	*ICD-10-CM or ICD-10-PCS code*
Dehiscence	0WQFXZZ, T81.30XA, T81.31XA, T81.32XA, T81.32
Infection	T81.49XA, T81.44XA, T81.40, T81.40XA, T81.41, T81.41XA, T81.42, T81.42XA, T81.43, T81.43XA, T81.44, T81.44XA, T81.49, T81.49XA, K65.1, K68.1, K68.11, K68.19, K68.9
Mortality	R99
Other	K91.72, K91.89, K65.0, K65.8, K65.9, J80
Stricture	K91.3, K91.31, K91.32
Stoma	0WBFXZ2, 0WQFXZ2
Revision	0DQ80ZZ, 0DQ83ZZ, 0DQ84ZZ, 0DQ87ZZ, 0DQ88ZZ, 0DQB0ZZ, 0DQB3ZZ, 0DQB4ZZ, 0DQB7ZZ, 0DQB8ZZ, 0DQE0ZZ, 0DQE3ZZ, 0DQE4ZZ, 0DQE7ZZ, 0DQE8ZZ
Shock	Y69, T81.10, T81.10XA
Urinary Tract Infection	N39.0

*ICD-10-CM*, International Classification of Diseases, 10th Revision, Clinical Modification; *ICD-10-PCS*, International Classification of Diseases, 10th Revision, Procedure Coding System.
